# Supplementary material for: Drug Holidays and Overall Survival of Patients with Metastatic Colorectal Cancer
Source: Cancers (Basel). 2021 Jul 13;13(14):3504. doi: 10.3390/cancers13143504 (PMC8304309; doi:10.3390/cancers13143504)
Supplement: Supplementary file 1 [file cancers-13-03504-s001.zip › cancers-1247971-supplementary.pdf]

cancers

MDPI

Supplementary Materials

Drug Holidays and Overall Survival of Patients with Metastatic Colorectal Cancer

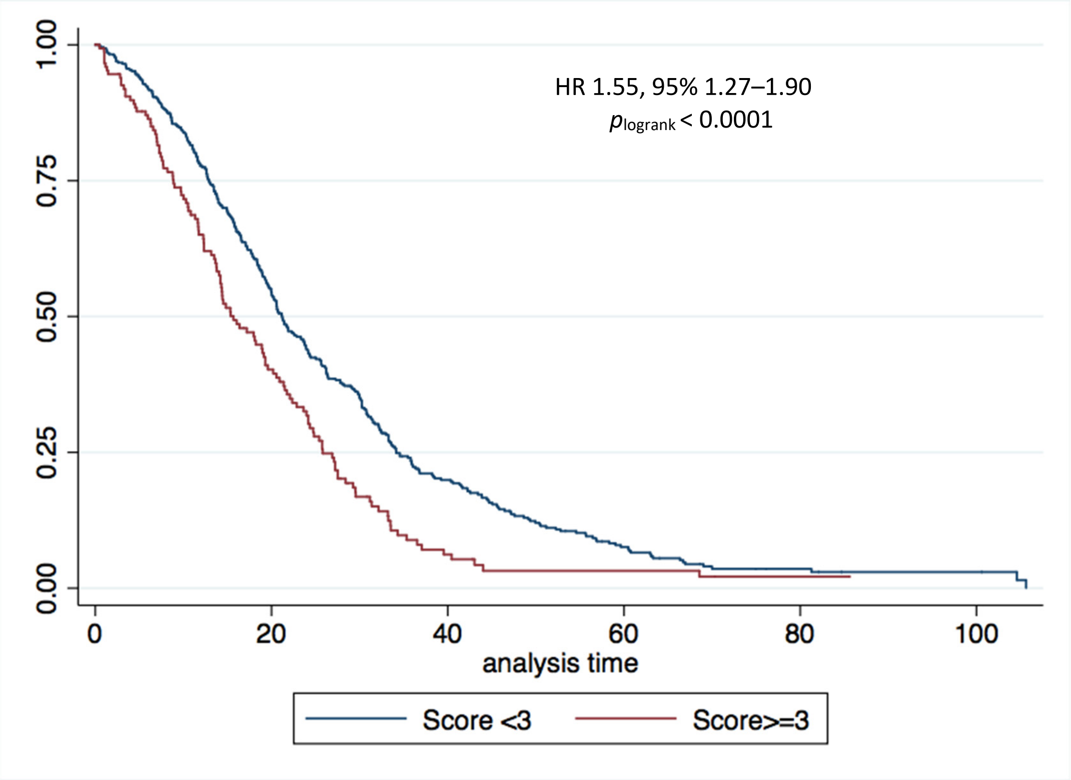

Figure S1. Kaplan-Meier survival curves according to cut-off.
